# Potent Antiproliferative Cembrenoids Accumulate in Tobacco upon Infection with *Rhodococcus fascians* and Trigger Unusual Microtubule Dynamics in Human Glioblastoma Cells

**DOI:** 10.1371/journal.pone.0077529

**Published:** 2013-10-22

**Authors:** Aminata P. Nacoulma, Veronique Megalizzi, Laurent R. Pottier, Manuela De Lorenzi, Sylviane Thoret, Joëlle Dubois, Olivier M. Vandeputte, Pierre Duez, Danny Vereecke, Mondher El Jaziri

**Affiliations:** 1 Laboratoire de Toxicologie, Faculté de Pharmacie, Université Libre de Bruxelles, Brussels, Belgium; 2 Laboratoire de Pharmacognosie, de Bromatologie et de Nutrition Humaine, Faculté de Pharmacie, Université Libre de Bruxelles, Brussels, Belgium; 3 Institut de Chimie des Substances Naturelles, Centre national de la recherche scientifique Unité PR 2301, Gif-sur-Yvette, France; 4 Laboratoire de Biotechnologie Végétale, Faculté des Sciences, Université Libre de Bruxelles, Gosselies, Belgium; 5 Department of Plant Production, University College Ghent, Ghent University, Gent, Belgium; Duke University Medical Center, United States of America

## Abstract

**Aims:**

Though plant metabolic changes are known to occur during interactions with bacteria, these were rarely challenged for pharmacologically active compounds suitable for further drug development. Here, the occurrence of specific chemicals with antiproliferative activity against human cancer cell lines was evidenced in hyperplasia (leafy galls) induced when plants interact with particular phytopathogens, such as the Actinomycete *Rhodococcus fascians*.

**Methods:**

We examined leafy galls fraction F3.1.1 on cell proliferation, cell division and cytoskeletal disorganization of human cancer cell lines using time-lapse videomicroscopy imaging, combined with flow cytometry and immunofluorescence analysis. We determined the F3.1.1-fraction composition by gas chromatography coupled to mass spectrometry.

**Results:**

The leafy galls induced on tobacco by *R. fascians* yielded fraction F3.1.1 which inhibited proliferation of glioblastoma U373 cells with an IC_50_ of 4.5 µg/mL, F.3.1.1 was shown to increase cell division duration, cause nuclear morphological deformations and cell enlargement, and, at higher concentrations, karyokinesis defects leading to polyploidization and apoptosis. F3.1.1 consisted of a mixture of isomers belonging to the cembrenoids. The cellular defects induced by F3.1.1 were caused by a peculiar cytoskeletal disorganization, with the occurrence of fragmented tubulin and strongly organized microtubule aggregates within the same cell. Colchicine, paclitaxel, and cembrene also affected U373 cell proliferation and karyokinesis, but the induced microtubule rearrangement was very different from that provoked by F3.1.1. Altogether our data indicate that the cembrenoid isomers in F3.1.1 have a unique mode of action and are able to simultaneously modulate microtubule polymerization and stability.

## Introduction

*Rhodococcus fascians* is a phytopathogenic Actinomycete that incites the development of so-called leafy galls (LGs) on a wide range of plant hosts. LG formation results from a reprogramming of plant cell fate and plant morphogenesis, leading to the *de novo* generation of shoot meristems and the activation of existing dormant shoot meristems (for a recent review see Stes *et al*., 2011). Although, a LG originates from the abundant proliferation of adventitious shoots, intriguingly, their further development is arrested soon after they are formed, unless the bacteria are killed [1]. LG are viewed as the ecological niche of *R. fascians* [2] and as plant structures where metabolism is modified to contribute to symptom development [1]. Phytohormones, mainly cytokinins but also auxins, produced by *R. fascians* are at the basis of symptom induction on the host plants, but the reason for the subsequent developmental blockage is currently unknown. LG can therefore be viewed as chemical interfaces where cell cycle activating and inhibiting compounds are produced.

Based on the knowledge that the hormone composition, but also the primary and secondary metabolism of the plant changes during LG development [1], we have previously demonstrated that particular LG-specific compounds might have a biological activities including antioxidant and anti-inflammatory activities [3]. LG tissues can also constitute a source of anti-tumoral compounds to reduce proliferation activity of human cancer cell lines [4]. The detected antiproliferative activity was investigated by analyzing the cell cycle duration, the cell growth, the ploidy levels, the occurrence of apoptosis, and the organization of the cytoskeleton. We identified the chemical nature of bioactive compounds that belongs to cembrenoid type diterpenes [4] and compared their activity to that of cembrene a commercially available and structurally related compound. Moreover, F3.1.1 showed marked effects on cytoskeletal architecture, where tubulin network is particularly altered. Cembrenoid structures might constitute a potential alternative for further development of new anti-tubulin drugs. Our results are discussed in the context of putative advances in anti-cancer therapeutics research

## Results and Discussion

### LG tissues contain potent compounds that affect the proliferation of different human cancer cell lines

**Figure 1:**
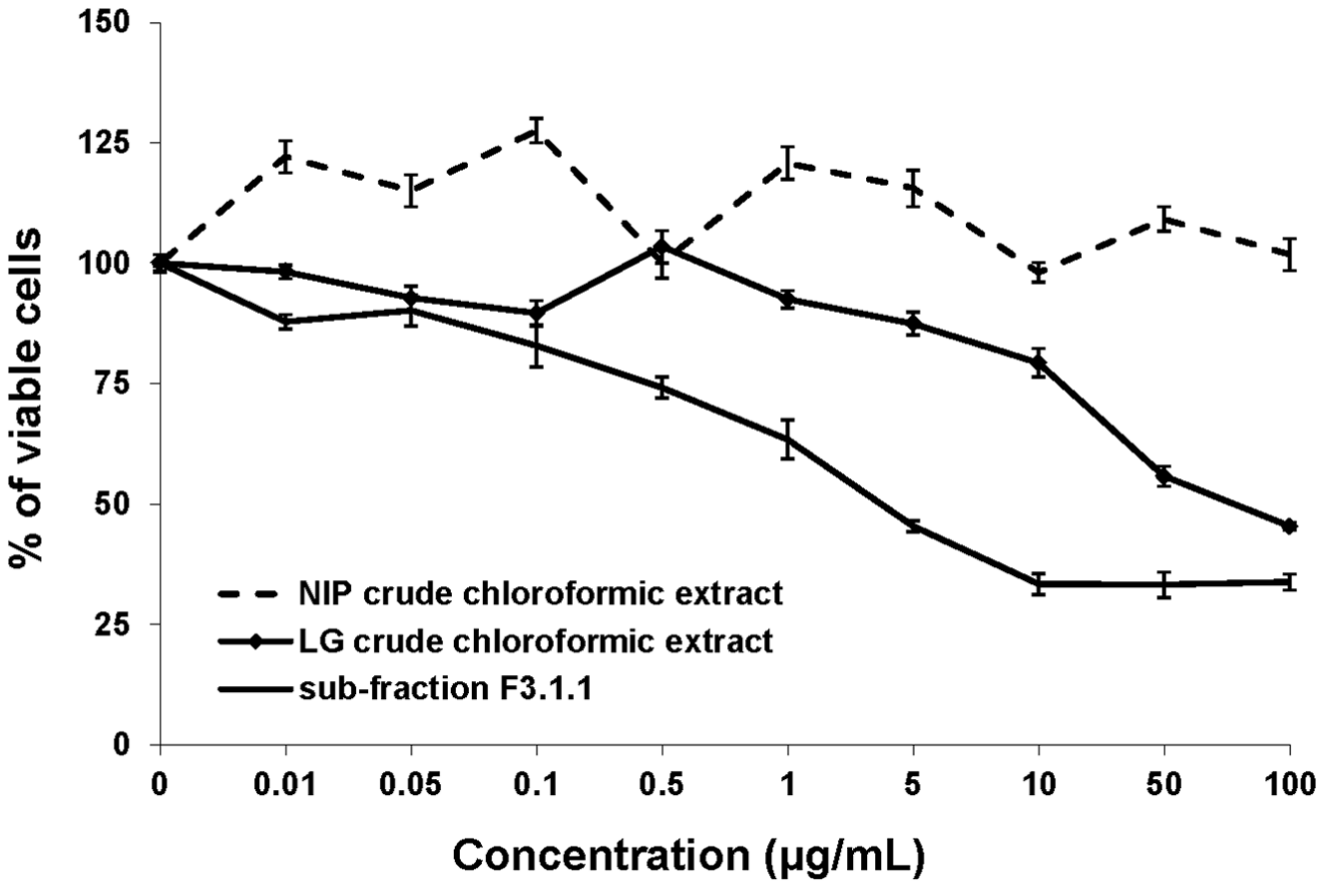
Crude chloroformic extract and sub-fraction F3.1.1. of *N. tabacum* (leafy galls (LG) and non-infected plants (NIP)) with antiproliferative activity against the glioblastoma U373 cell growth. Cells were treated for 72± SEM (n = 6).

The occurrence of antiproliferative activity in LGs and NIPs was previously assessed [4], and in our results mainly the chloroformic extract of LGs exhibited a distinct antiproliferative activity against all tested cell lines. A bio-guided fractionation of active sub-fraction of this crude chloroformic extract yielding to crystalized fraction F3.1.1 (7 mg), which proved to have an IC_50_ value of 4.5±0.3 µg/mL (Figure 1).

### The antiproliferative activity of F3.1.1 is caused by its cytostatic effect increasing the cell division duration

**Figure 2:**
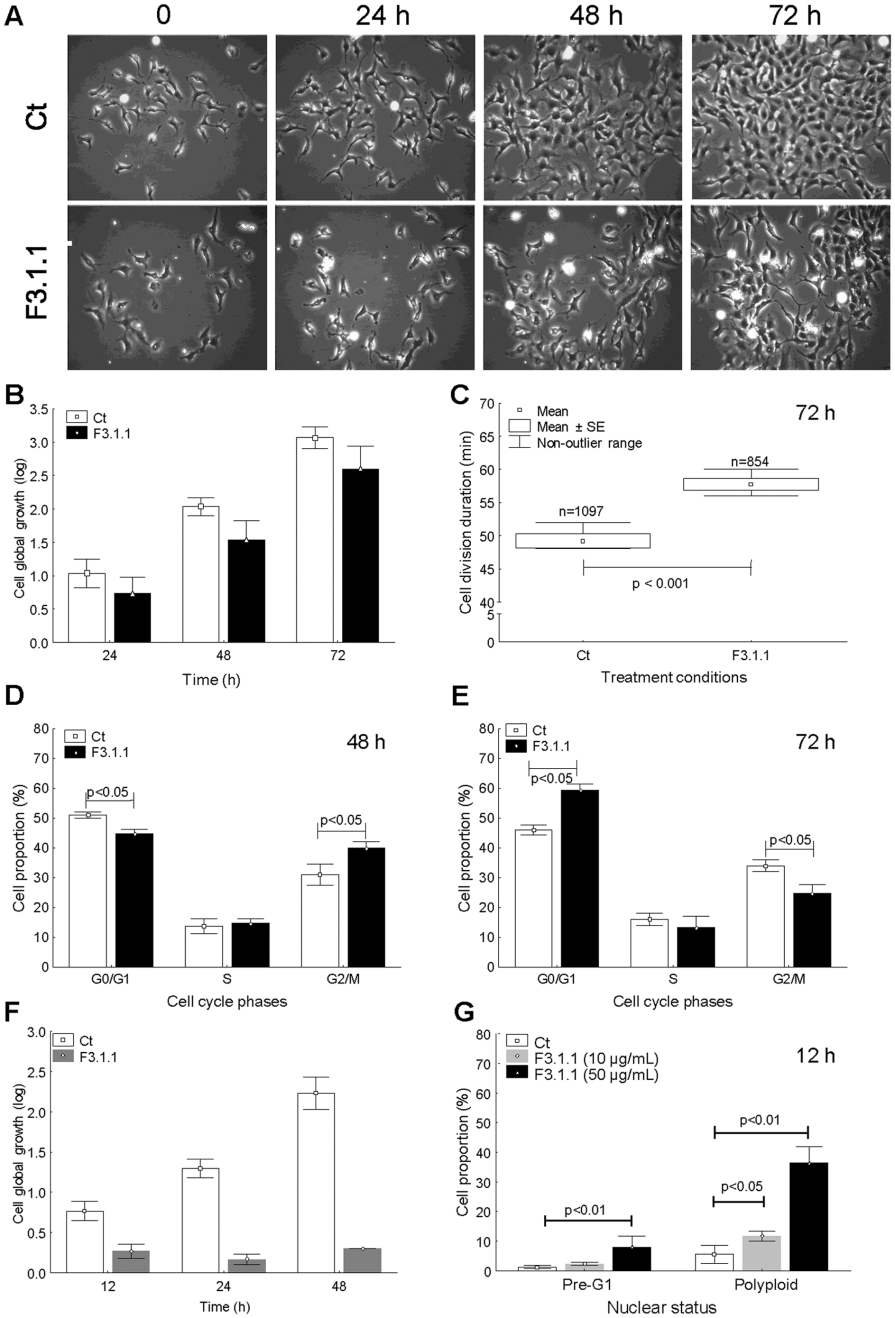
Analysis of cell division-related processes in control (Ct) and F3.1.1-treated U373 cells. Treatments with F3.1.1 were done at 4 µg/mL in a–e. (A) *In vitro* cell imaging by video microscopy after 24 h, 48 h and 72 h of growth. (B) *In vitro* cell global growth following 24 h, 48 h and 72 h of culture. The represented data are the mean ± SEM (n = 6). (C) Cell division duration over a time period of 72 h of growth in control (Ct) and treated cells (F.3.1.1). (D, E) Cell proportion in the different cell cycle phases at 48 h (D) and 72 h (E) for control (Ct) and F3.1.1-treated cells. The represented data are the mean ± SD (n = 3). (F) *In vitro* cell global growth at 12 h, 24 h, and 48 h of incubation with 10 µg/mL F3.1.1. The data represented are the mean ± SD (n = 3). (G) The occurrence of apoptopic cells (pre-G1 phase) and polyploid cells after 12 h of growth on 10 µg/mL or 50 µg/mL of F3.1.1. The represented data are the mean ± SD (n = 4).

To get insight into its mode of action, control and F3.1.1-treated (4 µg/mL≈IC_50_) U373 cells were imaged by videomicroscopy at different time points over a period of 72 h (Figure 2A), permitting the extraction of information on several aspects of cell division [5], [6]. As shown in Figure 2B, the cell proliferation rate, measured as cell global growth, increased steadily with time, but, compared to the controls, that of F3.1.1-treated cells was 20% lower after 24 h and 48 h, and 25% lower after 72 h. Because dividing cells can easily be distinguished from non-dividing ones by their bright and round appearance (Figure 2A), it is possible to deduce the cell division duration from the *in vitro* cell imaging [5], [6]. As shown in Figure 2C, the average duration of a cell division over a period of 72 h of F3.1.1-treated cells was 9 min longer than that of control cells. Together these observations confirm the antiproliferative activity of fraction F3.1.1 on U373 cells and indicate that it has a cytostatic effect, although it cannot be ruled out that F3.1.1 also exhibits some cytotoxicity.

Furthermore, the impact of F3.1.1 at 4 µg/mL on the cell cycle of U373 cells was analyzed by flow cytometry. After 24 h of growth, no differences were observed between F3.1.1-treated and control cells (Figure S1A). However, after 48 h of growth, compared to the control, the proportion of cells in the G2/M phase was 10% higher in F3.1.1-treated cultures (Figure 2D) and after 72 h, 60% of the F3.1.1-treated cells were in the G0/G1 phase, compared to only 45% of the control cells (Figure 2E). Altogether, these data indicate that F3.1.1 hampers or delays the progression of the U373 cell population through the cell cycle, which supports the observed increase in cell division duration (Figure 2C).

Finally, to establish whether F3.1.1 functions cytostatic rather than cytotoxic, cell apoptosis was evaluated. Therefore, U373 cells were treated with 4-IBP, a known inducer of apoptosis, or with 4 µg/mL F3.1.1 for 72 h and then the cell cycle phase distribution was analyzed by flow cytometry (see Methods). No pre-G1 population, indicative for apoptosis, was detected in F3.1.1-treated cells after 72 h, whereas 19% of the 4-IBP-treated cells were in this phase (Figure S1B). This observation clearly shows that at 4 µg/mL F3.1.1 has a cytostatic and no cytotoxic effect on U373 cells. When the concentration of F3.1.1 was increased to 10 µg/mL (≈2×IC_50_), its antiproliferative activity was already clear after 12 h of growth, no increase in cell proliferation was recorded with time, and the global growth rate after 48 h was 80% lower than that of the control (Figure 2F). However, with increasing concentrations, F3.1.1 also affected the ploidy level of the U373 cells. Already after 12 h of growth with 10 µg/mL of F3.1.1, the fraction of polyploid cells doubled compared to the control and upon incubation with 50 µg/mL (≈10×IC_50_), this even increased to 31% (Figures 2G and S2). Moreover, flow cytometric quantification of the proportion of cells in the pre-G1 phase showed that whereas 10 µg/mL of F3.1.1 did not cause apoptosis, a concentration of 50 µg/mL did exhibit some level of cytotoxicity (Figures 2G and S2).

### Fraction F3.1.1 contains a mixture of diterpenes belonging to the cembrenoid family

**Figure 3:**
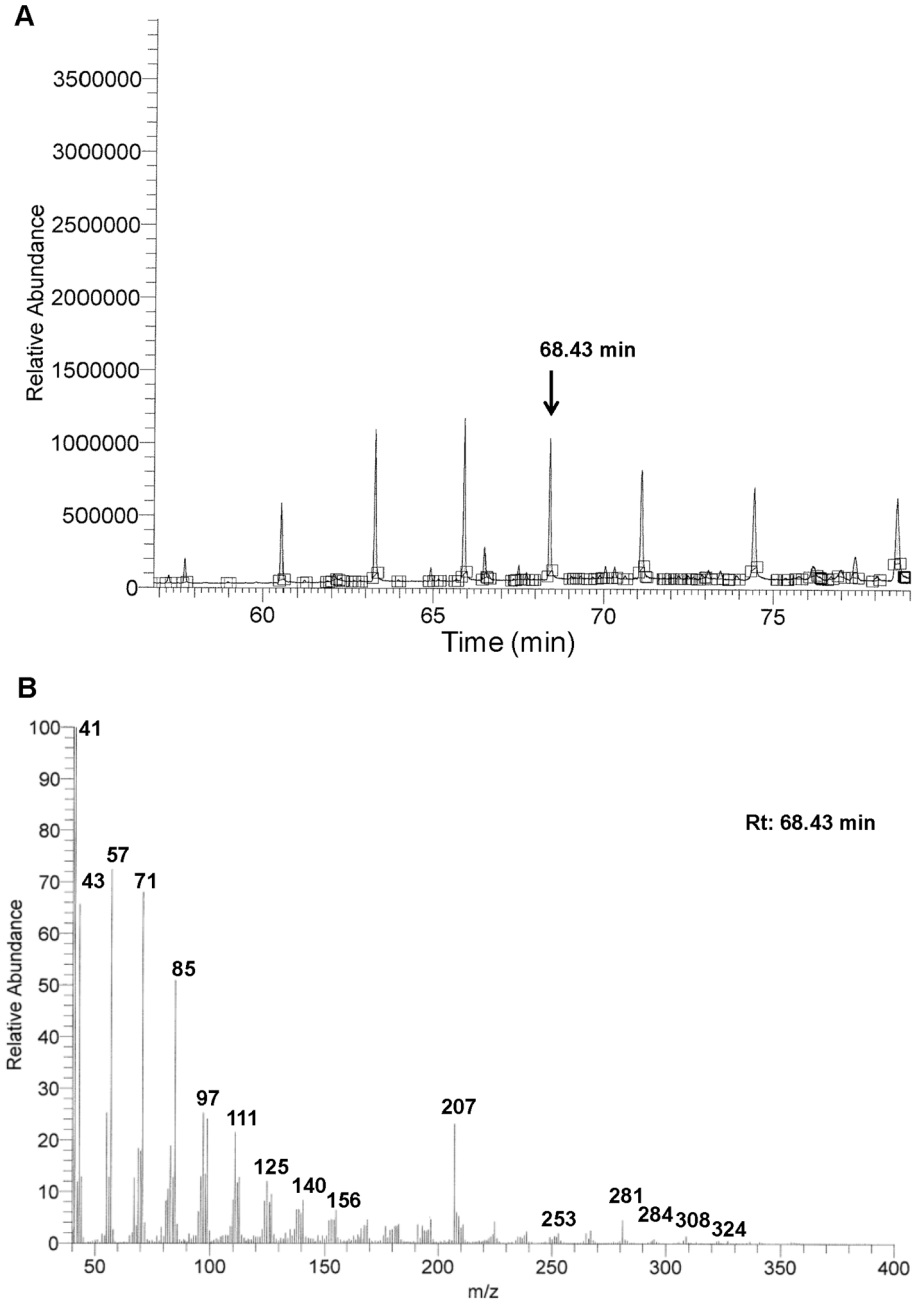
GC-MS analysis of fraction F3.1.1. (A) GC chromatogram of fraction F3.1.1. (B) Mass spectrum of the peak eluted at 68.43 min.

To elucidate the identity of the bioactive compound, fraction F3.1.1 was submitted to GC-MS and ^1^H NMR analysis. The GC chromatogram revealed that F3.1.1 consisted of a mixture of ten compounds with different retention times (Figure 3A). Unexpectedly, the mass spectra recorded for each of the peaks in the spectrum was almost identical, albeit that the relative abundance of the mass signals slightly differed for each compound (Table S1). Typically, such GC-MS results indicate that the compounds in F3.1.1 are closely related isomers, explaining the spontaneous co-crystallization observed in fraction F3.1 (Figure 1A). Previously, by mean of, exploratory ^1^H NMR-based metabolomics and GC-MS analyses of total chloroformic LG and NIP extracts under particular chromatographic conditions revealed that LGs were highly enriched in diterpenoids, more specifically cembrenoids [4]. Based on these data we postulated that the compounds in F3.1.1 could be macrocyclic diterpenes of the cembrenoid family [7]–[10].

**Figure 4:**
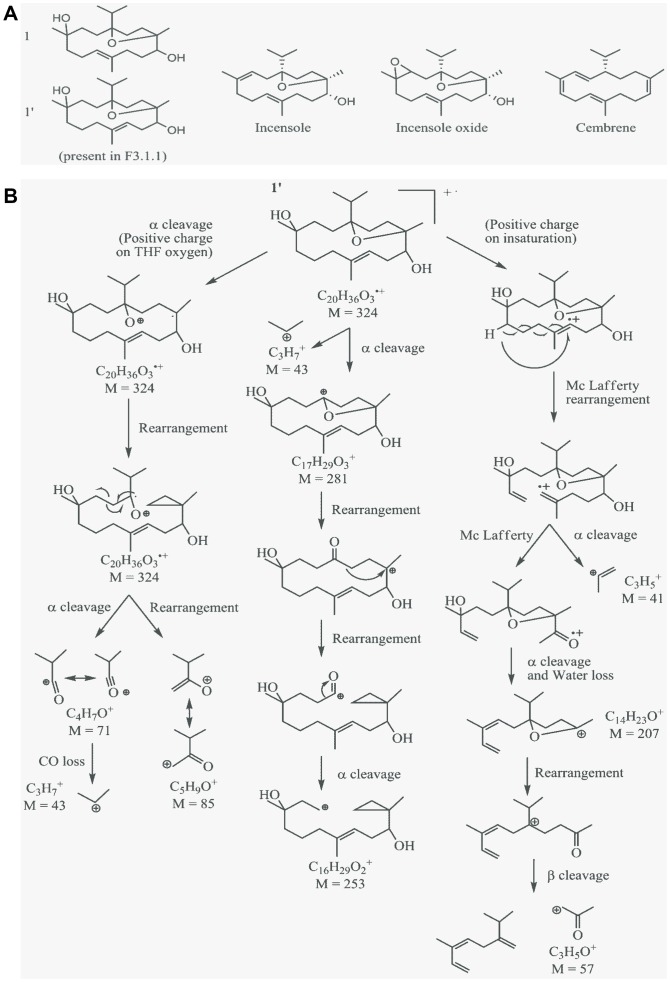
Chemical structure of known cembrenoids and proposed structure of the compounds in F3.1.1. (A) Structure of incensole, incensole oxide and cembrene. (B) Hypothetical structure **1′** and three possible routes of its fragmentation. The structure is proposed based on ^1^H NMR and the mass spectrum (EI 70 eV) of the compounds present in F3.1.1.

Indeed, cautious examination of the fragmentation pattern of the peak at retention time 68.43 min (Figure 3B) and comparison with those of cembrene, incensole and incensole-oxide (Table S2; Figure 4A), suggested that it belonged to the incensole family. Analysis of its principal mass fragmentation pattern combined with the predicted presence of a double bonds at δ 5.35 ppm and isopropyl groups at δ 1.27 ppm in ^1^H ratio 1∶6 by ^1^H NMR of the F3.1.1 mixture (Figure S3), allowed the proposition of putative structures containing 1 double bound for 1 isopropyl group (designated structures **1** and **1′**; Figure 4B). The high similarity in the fragmentation patterns for all the compounds in fraction F.3.1.1 (Table S1), implies that they are close isomers of structure **1** that only differ from each other in the position of the double bond or in the allocation of the hydroxy groups.

Cembrenoids are commonly found in the plant and animal kingdoms [9]. They are known inhibitors of plant growth and fungal spore germination and are reported to have insecticide, antimicrobial, antioxidant and anticancer activities. [9]–[11]. In tobacco, cembrenoid biosynthesis is confined to trichome glands [12], [13]. Accordingly and since LG is a compact structure with multiple malformed leaves with abundant thrichomes [1], one can assume that the antiproliferative activity recorded in LG chloroformic extracts may be due to cembrenoids accumulated in trichome structures. However, it cannot be ruled out that ectopic expression of the cembrenoid biosynthetic pathway occurs upon infection with *R*. *fascians*.

**Figure 5:**
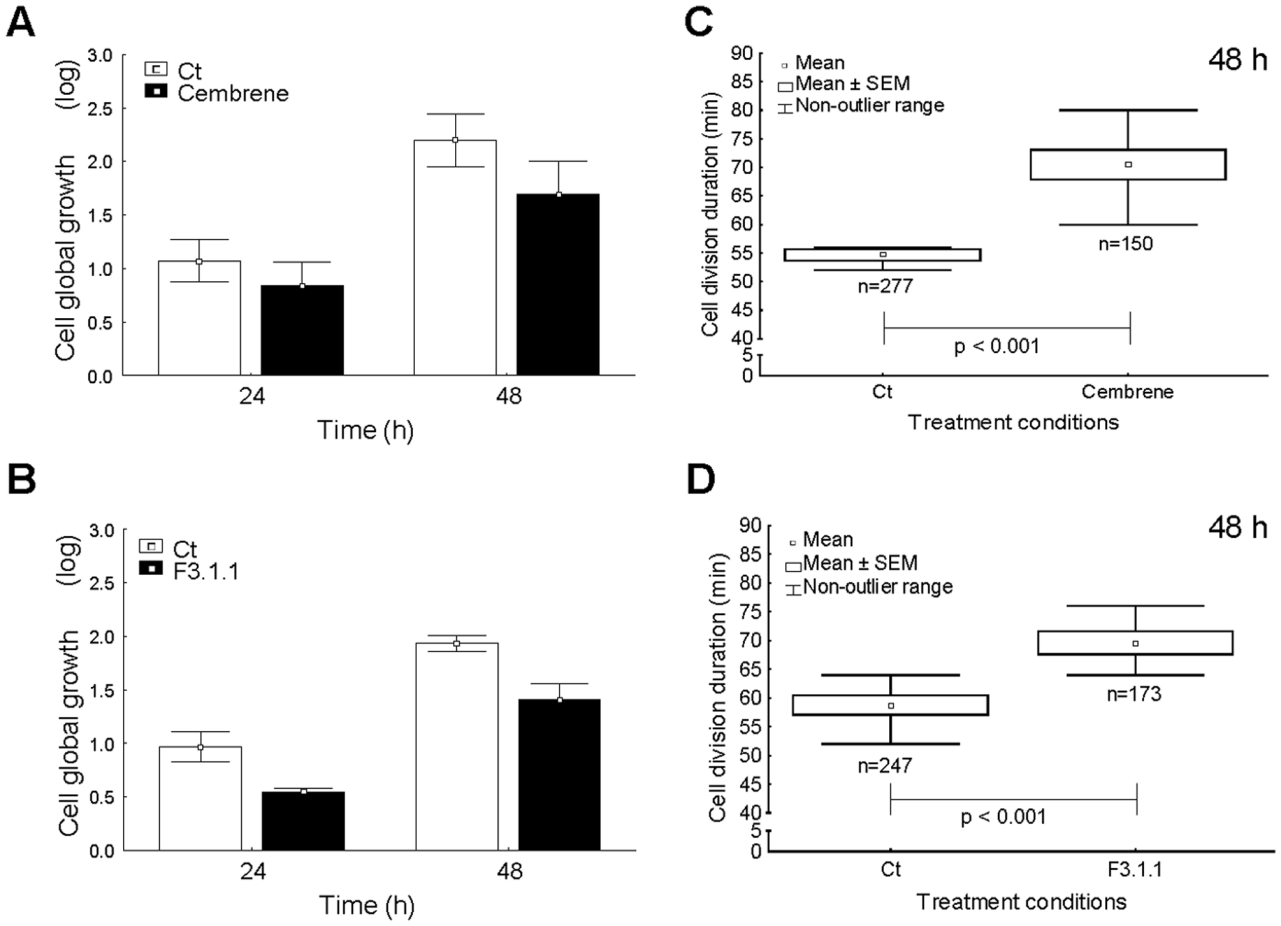
Analysis of cell division-related processes in control (Ct), F3.1.1-, and cembrene-treated U373 cells. Cells were treated with 4 µg/mL of F3.1.1 or cembrene. (A, B) *In vitro* cell global growth after 24 and 48 h of culture of (A) cembrene- and (B) F3.1.1-treated cells. (C, D) Cell division duration over a time period of 48 h of growth in (C) cembrene- and (D) F.3.1.1- treated cells. The data are represented as the mean ± SD (n = 3).

To test whether the biological activity of F3.1.1 could be attributed to the presence of cembrenoids, the effect of cembrene, the only commercially available cembrenoid, on U373 cells was assessed. First, the IC_50_ of cembrene for cell proliferation was determined by MTT assay means to be 4 µg/mL (data not shown) and then, based on phase-contrast time-lapse image series, cell proliferation and cell division duration were analyzed in more detail. At the IC_50_, compared to the control, the proliferation rate of cembrene-treated cells was reduced by 20% and 30% after 24 h and 48 h of growth, respectively (Figure 5A), whereas cells treated with F3.1.1 at its IC_50_ exhibited 44% and 30% lower proliferation rates at these time points (Figure 5B). Deduction of the average cell division duration time showed that compared to the control, cembrene prolonged a cell division event by 15 min over a 48 h period of growth (Figure 5C), whereas this was 12 min for F3.1.1 treatment (Figure 5D). Thus, the effects of cembrene and F3.1.1 are largely comparable.

### F3.1.1 affects the tubulin/microtubule dynamics in a very peculiar fashion different from that of colchicine, paclitaxel and cembrene

**Figure 6:**
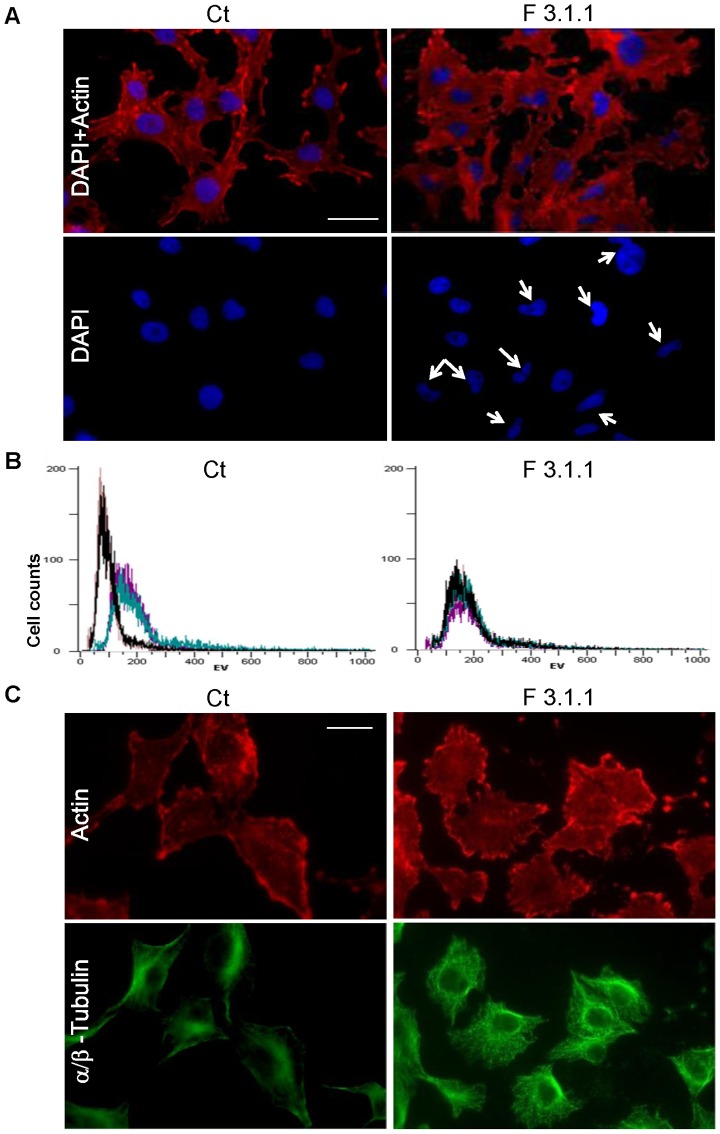
Cytological analysis of control (Ct) and F3.1.1-treated U373 cells. All treatments with fraction F3.1.1 were done at 4 µg/mL for 72 h. (A) F-actin filament immunostaining with phalloidin conjugated to Alexa Fluor 488 (red) and visualization of nuclei with DAPI staining (blue). Arrows indicate irregularly shaped nuclei. (B) Flow cytometry analysis of the cell size distribution of the entire cell population as measured by electronic volume (EV). (C) Cytoskeleton immunostaining for F-actin (red) and with anti-α/β-tubulin antibodies for the microtubule arrangement (green). The scale is the same in all panels (bar  = 20 µm).

To understand the cause of the cytostatic effect of F3.1.1 at 4 µg/mL, the cytoskeleton and nuclei of F3.1.1-treated and control U373 cells were visualized *via* immuno- and DAPI-staining, respectively. Although no clear differences were observed in the actin network, F3.1.1 treatment did seem to affect cell shape and size (Figure 6A). The latter finding was confirmed by flow cytometry using electronic volume (EV) as a measure. The control cells showed two distinct sub-populations (90 and 150 EV), while the entire population upon treatment with F3.1.1 shifted to the largest cell size (Figure 6B).

At 4 µg/mL, F3.1.1 also affected the nuclear morphology. Whereas control cells had smooth and round or oval nuclei, in F3.1.1-treated cells irregularly shaped nuclei occurred frequently (Figure 6A). Moreover, the occasional observation of nuclei linked by chromatin bridges in the treated cells indicated that F3.1.1 might interfere with the dynamics of the mitotic spindle thus affecting karyokinesis and resulting in incomplete cell segregation [14] Analysis of the videomicroscopy image sequences allows the get information on the different steps culminating in cytokinesis, including karyokinesis (Figure S4A). Careful examination of these images of F3.1.1-treated cells clearly demonstrated the occurrence of karyokinesis defects (Figure S4B) and quantification of the karyokinesis duration time revealed that this process was extended by 6.5 min compared to the control (Figure S4C). Altogether these data imply that the tubulin/microtubule organization might be the target of the cembrenoid compounds present in F3.1.1.

Therefore, the microtubule network was examined by α/β-tubulin immunostaining. The control cells exhibited a homogenous and rather diffuse staining, but, in the F3.1.1-treated cells the immunostaining occurred both in dots and around nuclei in bright foci, signifying the simultaneous occurrence of fragmented tubulin and organized microtubules, respectively, in single cells (Figure 6C). These findings suggest a profound and unique alteration in the tubulin/microtubule dynamics, which explains the cytotoxicity of F3.1.1 at 50 µg/mL and the higher proportion of polyploid cells in these treated U373 populations (Figure 2G). Indeed, microtubule stability is known to determine cell death after mitotic exit [15], [16]. Accordingly, it is tempting to postulate that the sub-population of F3.1.1-treated cells that cannot form a mitotic spindle because of the disrupted microtubules possibly escape mitosis reconstituting a tri- or tetraploid G1 population, whereas other cells likely remain in mitotic arrest leading to an obligatory entry into apoptosis.

**Figure 7:**
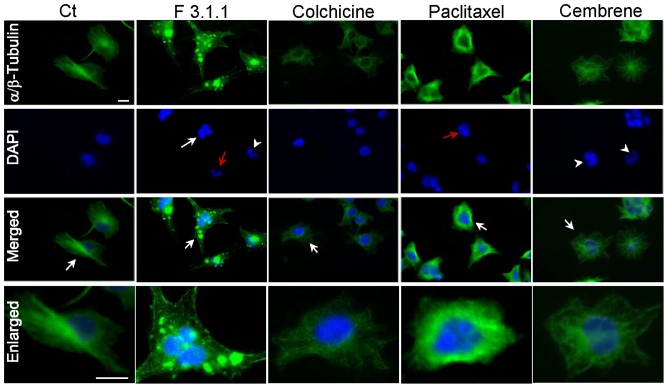
Tubulin and nuclei visualization in U373 cells treated with F3.1.1 (10 µg/mL), colchicine (16 nM), paclitaxel (13 nM) or cembrene (8 µg/mL). In the DAPI panel of the F3.1.1, paclitaxel or cembrene-treated cells, the red arrow indicates a nucleus that is deteriorated to micronuclei, the arrowhead points to two nuclei linked by a chromatin bridge, and the arrow to the nucleus of a polyploid cell. In the panels with the merged pictures, the arrows show the cells that are enlarged in the lower panels. Bar  = 20 µm.

To get insight into its mechanism of action, the effect of F3.1.1 on the cytology of U373 cells was compared to that of colchicine and paclitaxel, two cytotoxic compounds known to alter cell division by inhibiting α/β-tubulin polymerization [16], [17] or by promoting microtubule assembly [16], respectively. Treatment of U373 cells for 24 h with 10 µg/mL of F3.1.1 (≈2×IC_50_) caused a more pronounced effect on the nuclear morphology than obtained at the IC_50_. Indeed, a defective karyokinesis was evident not only by the occurrence of nuclei linked by chromatin bridges resulting in polyploid cells, but also of nuclei that deteriorated to micronuclei (Figure 7). These nuclear defects were associated with a severe disorganization of the microtubule network, which consisted of both fragmented tubulin and densely organized microtubules localized in multiple aggregates dispersed throughout the cell (Figure 7). Colchicine treatment at 2×IC_50_ for 24 h did not cause any apparent nuclear damage, but a comparable treatment of the U373 cells with paclitaxel resulted in nuclei linked by chromatin bridges, micronuclei and polyploidy, similar to what was observed with the higher F3.1.1 concentrations (Figure 7). Interestingly, the effect of colchicine and paclitaxel treatment on the cytoskeleton was not comparable to that induced by F3.1.1. Whereas colchicine caused a complete depolymerization of the microtubules, paclitaxel treatment lead to an extensive tubulin polymerization around the centrosome (Figure 7). Thus, the cytology of F3.1.1-treated U373 cells suggests a combined effect of colchicine and paclitaxel, leading simultaneously to the inhibition α/β-tubulin polymerization and the interference with microtubule depolymerization. However, the microtubule arrangement obtained after F3.1.1 treatment also differed significantly from that reported for cells treated with colchitaxel, a synthesized compound coupling colchicine and paclitaxel structures, or with mixtures of different concentrations of colchicine and paclitaxel [18]. Thus, based on these data, it seems unlikely that the particular effect caused by F3.1.1 treatment on U373 cells results from the fact that it is a mixture of compounds, rather F3.1.1 appears to have a different mode of action than colchicine and paclitaxel. The effects of F3.1.1 previously described on U373 cells are similar to those observed on another glioblastoma cell line (T98G). In Figure S5, F3.1.1-treated cells show irregularly shaped nuclei, microtubule disorganization with both fragmented tubulin and densely organized microtubules localized on the centrosome or in multiple aggregates dispersed throughout the cell.

**Figure 8:**
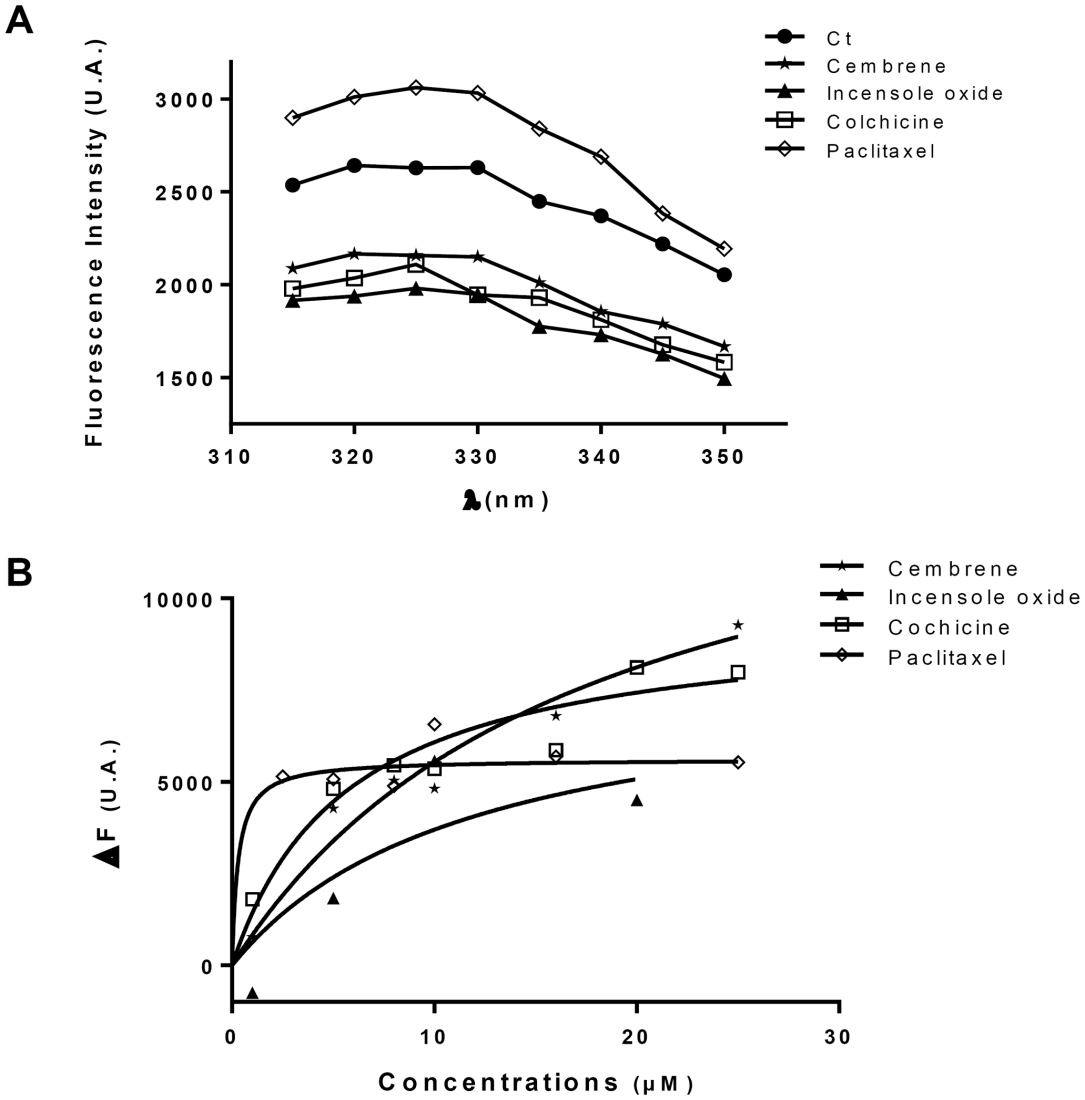
Reduction of intrinsic tryptophan of tubulin by cembrenoids and determination of dissociation constant. (A) The effects of cembrenoids on the tryptophan fluorescence spectra of tubulin are shown. Spectra are monitored in the absence (Ct) and presence of 5 µM of Cembrene, Incensole oxide, Colchicine or Paclitaxel (B) The change in the fluorescence intensity of tubulin (ΔF) is plotted against concentrations of each compound. The dissociation constant (Kd) for Cembrene, Incensole oxide, Colchicine and Paclitaxel binding to tubulin is estimated using an equation described in the methods. Data are represented as the mean ± MAD of two independent experiments.

Finally, the effect of F3.1.1 was compared to that of cembrene. Recent *in silico* conformational analysis suggested that cembrene could dock both into the paclitaxel and the colchicine binding sites of tubulin, although the predicted affinity for the colchicine binding site was greater [19]. Experimental support for this prediction came from *in vitro* tubulin polymerization assays using purified sheep brain tubulin. Because cembrene has a very low solubility (estimated LogP∼7 by ALOGPS 2.1), we could only assess its inhibition of tubulin polymerization (ITP) for a maximum concentration of 3.3 µg/mL. Although cembrene did not inhibit tubulin polymerization at 0.6 µg/mL, at 3.3 µg/mL its ITP activity was in the same range as that of colchicine, 33% and 40%, respectively (Table 1). Then, the effect of cembrene treatment at 8 µg/mL, (2×IC_50_) for a 24 h-period on U373 cells was analyzed in greater detail. Unlike colchicine, but similar to paclitaxel and F3.1.1, the cembrene-treated cells showed nuclei linked by chromatin bridges leading to polyploidy (Figure 7). Moreover, cembrene treatment prolonged the karyokinesis duration time by 3 min compared to control cells (Figure S4C). Just like F3.1.1, cembrene did not affect the actin network (Figure S6), but it did modify the cell shape (Figure 7). Although cembrene caused fragmentation of the microtubule network, comparable to that observed with colchicine, it did not provoke localized microtubule aggregations as obtained by F3.1.1 treatment (Figure 7). In order to confirm tubulin-binding affinity of cembrenoids, we performed Tryptophan Fluorescence of Tubulin assay. Cembrenoids reduced the intrinsic tryptophan fluorescence intensity of tubulin in a concentration dependent manner (Figure 8A). Indeed, in the presence of cembrenoids at 5 µM the reduction of tryptophan fluorescence of tubulin are reached of 18% for cembrene and 25% for incensole oxide. Fitting the fluorescence changes gives a binding Kd of 22.2±4.8 µM and 9.8±2.2 µM for cembrene and incensole oxide respectively when compared to colchicine (7.6±0.6 µM) and paclitaxel (0.5±0.2 µM) (Figure 8B).

**Table 1:** Inhibition of Tubulin Polymerization (ITP) assay with cembrene, colchicine and paclitaxel.

Compounds	Tested concentrations (µg/mL)	ITP activity (%)
Cembrene	0.6	0
	3.3	33
Colchicine	0.6	25
	3.3	40
	6.6	66
Paclitaxel	5.7	0

Altogether, these results indicate that although F3.1.1, colchicine, paclitaxel and cembrene have partially overlapping effects on the behavior of U373 cells, importantly, the simultaneous occurrence in a single cell of fragmented microtubules and aggregates of polymerized tubulin seems to be specific for F3.1.1. Thus, F3.1.1 apparently combines the activities of a depolymerizing and a stabilizing agent into a single molecule. We speculate that this particular effect of F3.1.1 results from its equal affinity for both the colchicine and the paclitaxel binding sites of tubulin.

## Conclusions

The importance of microtubules in cell division but also in organelle transport, cell shape maintenance and motility, make them important targets for cancer drugs. For instance, agents that affect microtubule depolymerization, such as taxoids (paclitaxel and docetaxel), are currently used with positive results in ovarian, breast and non-small cell lung cancer therapies [20]. However, the development of resistance in tumors to tubulin-binding agents, such as paclitaxel, caused by mutations in β-tubulin is one of the most significant obstacles for successful treatment [21]. Therefore, the identification of new molecules, such as the cembrenoids in fraction F3.1.1, that target the microtubule dynamics by a different mechanism could provide a better understanding of the molecular basis of resistance to tubulin-binding agents and importantly, may contribute to the improvement of therapies targeting microtubule organization.

The unique features of the LG cembrenoids and their peculiar mode of action on the tubulin/microtubule dynamics illustrate that plant pathologies are worth exploring for novel potential anti-cancer agents.

## Methods

### Plant material and bacterial infection

The growth conditions for *R. fascians* strain D188 and tobacco plants (*Nicotiana tabacum L*. cv. Petit Havana), as well as the infection procedure of 4-week-old *in vitro* tobacco plantlets were done as described by Rajaonson *et al.* (2011) [22]. Developed leafy galls (LG) were harvested eight weeks after infection; non-infected plants (NIPs) after twelve weeks of growth.

### Extraction of plant material

The chloroformic dry crude extract (2320 mg), obtained from 1.03 kg of LG material, was subjected to two successive automated flash chromatography systems (CombiFlash® Rf 200 psi from Teledyne isco®, Lincoln, USA). The column was filled with a normal phase silica gel (4 g silica RediSep® Rf columns from Teledyne isco®, Lincoln, USA) using a binary gradient mobile phase composed of dichloromethane:methanol (from 0% to 40% of methanol) at a flow rate of 15 mL/min and a second mobile phase composed of dichloromethane:methanol (from 10% to 30% methanol) at a flow rate of 10 mL/min and flash chromatography are performed to give sub-fractions among which sub-fraction F3.1 is crystallized in methanol at 4°C giving 7 mg of sub-fraction F3.1.1.

### Established cell lines

The human U373 (ATCC code HTB-17) and T98G (CRL-1690) glioblastoma cancer cell lines were obtained from the American Type Culture Collection (ATCC; Manassas, VA, USA) and maintained in our laboratory as described previously [5].

### Samples preparation for proliferation bioassay

For proliferation bioassay, LG and NIP extracts as well as the chromatographic fractions were dissolved at a concentration of 1 mg/ml in DMSO and adjusted to 10% DMSO (v/v) with culture medium before to be subjected to proliferation inhibition assay at appropriate concentrations of the tested samples. The final concentration of DMSO in the cell cultures was 1% (v/v). For the control treatment, DMSO 1% (v/v) was used. All tested cancer cell lines have been previously cultured in medium supplemented with DMSO 5% (v/v, final concentration) without any modifications in their growth levels when compared to cells cultured in DMSO-free medium (data not shown).

### Cell proliferation bioassay

The colorimetric MTT viability assay (3-(4,5-dimethylthiazol-2-yl)-2,5 diphenyltetrazolium bromide; Sigma, Belgium) was used to determine the overall growth level of each cell line at 72 h as described previously [23] (n = 6 for each concentration).

### Video microscopy

A phase-contrast video microscopy analysis was performed as previously described.[5].

### Determination of global growth rate and cell division duration

Based on the image sequences acquired *via* videomicroscopy from control and treated cell cultures after 24, 48 or 72 h of growth, the global growth rate was calculated between the number of cells in the last and first frame of the image sequences. All cell counts were done in triplicate or sextuplicate using an interactive computer tool [5].

The same set of images was used to evaluate the cell division duration. With a custom division detector developed to measure the duration of cellular divisions over a 48 h or 72 h period. Therefore, global cell counts on different images extracted from time-lapse sequences and which provided the number of cell division that had occurred after a time-period in the different treatments [5]. All experiments were performed in triplicate or sextuplicate.

### Flow cytometric analysis of the cell cycle phases distribution and cell size distribution in U373 cell cultures

Cells were washed in phosphate-buffered saline (PBS) and fixed in cold ethanol 70% (v/v) in PBS for at least 24 h at −20°C. Fixed cells were washed with PBS and stained with a PBS solution containing propidium iodine (PI) (50 µg/mL) and RNAse (1 mg/mL) for 30 min, in the dark at 37°C. Subsequently, the cells were incubated at 4°C and the DNA content was analyzed through a Cell Lab Quanta SC flow cytometer (Beckman Coulter, USA) [23]. For each condition analyzed, 10 000 cells were counted. All experiments were performed in triplicate or quadruplicate.

### Analyses of cytoskeleton organization and nuclear morphology

To display the cytoskeleton organization and the nuclear morphology, treated and control U373 cells were fixed 20 min with formaldehyde 4% (pH 7.6) on coverslips and immunostained: 2 h with anti-α/β-tubulin antibodies (Santa Cruz Biotechnology, at dilution 1/50), followed by 1 h incubation with a secondary antibody conjugated to the Alexa Fluor 594 fluorochrome, then 1 h with fluorescent phalloïdin conjugated to the Alexa Fluor 488 fluorochrome (Molecular Probes, Invitrogen), and finally with DAPI, visualizing respectively tubulin, actin, and DNA (nuclei). The coverslips were then mounted on microscope slides with 20 µL polyvinyl alcohol mounting medium containing DABCO®, an antifading agent (Fluka, Sigma-Aldrich, USA). Three coverslips per experimental condition were analyzed and three pictures were taken for each coverslip (with the same exposure time) using an AxioCamHRm fluorescent microscope (Zeiss, Germany).

### GC-MS analysis

GC–MS analyses were performed on a Thermo ITQ900 (Thermo Fisher Scientific Inc©, USA) mass spectrometer equipped with an electron-impact (EI) source used at 70 eV in the scan range m/z 29–400. The chromatography was done on a Rxi-5 Sil MS capillary column (Restek Corporation, USA) with helium as carrier gas at a column head pressure of 10 psi. The sample was introduced into the capillary column by splitless injection (2 µL of sample at 50 µg/mL, 1 min). The injector temperature was set to 250°C and the gradient temperature scheme was as follows: 40°C for 1 min, 9°C/min increase rate up to 130°C, and 2°C/min increase rate up to 230°C. The temperatures of the transfer line and the source were 250°C and 150°C, respectively.

### Tubulin polymerization inhibition assay

To measure the effect of cembrene, colchicine and paclitaxel on the tubulin assembly rate, appropriate quantities of the compounds were dissolved in DMSO and 1 µL thereof was added to 150 µL-aliquots of the tubulin solution heated to 37°C as previously described [24].

### Measurement of the dissociation constant of the binding of cembrenoids to tubulin using tryptophan fluorescence of tubulin

To measure the tubulin-binding of cembrene (commercially available) and incensole oxide (extracted from Boswellia species in our laboratory), tubulin (Cytoskeleton) (Tebu Bio, Boechout, Belgium) (2 µM) in 25 mM PIPES buffer pH 6.8 was incubated without and with different concentrations of cembrene or incensole oxide (an F3.1.1 analogue) at 25°C for 30 min, the paclitaxel and colchicine were used as references. The fluorescence intensity was monitored in 96 wells UltraCruz UV-plates (Santa Cruz Biotechnology, Huissen, Nederland) by exciting the reaction mixture at 295 nm and the emission spectrum was recorded with Synergy Mx (BioTek, Colmar, France) in the range of 315 nm to 350 nm. We selected 295 nm as the excitation wavelength to specifically excite the tubulin tryptophan residues. When excited at 295 nm, tubulin displayed atypical emission spectrum with a maximum at 325 nm [25].

Fluorescence data are fitted in the following equation: 
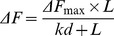



Where, ΔF is the change in the fluorescence intensity of tubulin in the presence of compounds, ΔF_max_ is the maximum change in the fluorescence intensity of tubulin when it is saturated with compounds and L is the concentration of compounds. The intrinsic fluorescence of paclitaxel was corrected and the dissociation constant (Kd) for cembrenoids and references binding to tubulin were estimated using the Graph Pad Prism 6 software (Graph Pad Software, CA, USA).

### Statistical analysis

The data obtained from independent groups were compared by the nonparametric Kruskal-Wallis test (more than two groups) and subsequently, control and treated conditions were compared using the nonparametric Mann–Whitney test (Statistica 7, StatSoft Inc., USA). For the cell global growth where the data are expressed as logarithmic values, graphical data were presented as the mean values ± SEM (n = 6) or SD (n<6).

## Supporting Information

Figure S1 **Flow cytometry analysis of the relative cell cycle phase distribution of treated and control U373 cell cultures.** (A) Control (Ct) or treated cells with F3.1.1 at 4 µg/mL (n = 3); (B) Apoptosis induced in cells treated with 4-IBP at 10 µM (n = 3).
(TIF)

Figure S2 **Flow cytometry analysis of U373 cells treated with higher concentrations of F3.1.1.** As compared to control cells (Ct), a complete cell cycle disruption is observed with 10 µg/mL and 50 µg/mL of F3.1.1. A concomitant increase in polyploidy is observed in cells treated with 50 µg/mL of F3.1.1 (overlap of four experiments n = 4).
(TIF)

Figure S3 **^1^H NMR spectrum of the F3.1.1 mixture.** Proton chemical shift at δ 5.35 ppm and δ 1.27 ppm correspond to double bonds and isopropyl groups respectively. ^1^H NMR (CDCl3, 500 MHz, pulse width of 5 µs (flip angle of 30°), scans number of 32, Bruker Avance II, TMS).
(TIF)

Figure S4 **Analysis of videomicroscopic image sequences and karyokinesis duration in control and treated U373 cells.** (A) Triplicate of a representative image sequence of a dividing control cell with the green squares in the thumbnails indicating that part of the sequence that identifies a particular event such as (1) karyokinesis, (2) division with well-defined daughters, and (3) cytokinesis. (B) Two image sequences illustrating karyokinesis defects in cembrenoid-treated cells with the green squares in the thumbnails indicating that part of the sequence that identifies (1) the inability to distinguish cytokinesis, and (2) the inability to distinguish two daughter cells due to asynchronous division. (C) Average cell karyokinesis duration over a time period of 48 h in control (Ct) and treated cells with 4 µg/mL cembrene or F.3.1.1. The number of cells undergoing karyokinesis is indicated (n). The statistical significance was evaluated as described in Methods.
(TIF)

Figure S5 **Actin, tubulin and DNA visualization in T98G cells upon treatment with 10 µg/mL F3.1.1.** Immunostaining of T98G cells untreated (Ct) and treated with F3.1.1 (2×IC50) for 24 h. Cells on coverslips were fixed and stained with anti-a/b-tubulin, followed by phalloidin-conjugated to Alexa Fluor 488 and finally with DAPI. Arrows show abnormal nuclei. Bar 20 µm, scale is the same in all panels.
(TIF)

Figure S6 **Actin, tubulin and DNA visualization in U373 cells upon treatment with 8 µg/mL cembrene.** Immunostaining of untreated (Ct) and treated cells (2×IC50) after 24 h of incubation. Cells on coverslips were fixed and stained with anti-α/β-tubulin antibodies for tubulin (green), followed by phalloidin conjugated to Alexa Fluor 488 for actin (red) and/or with DAPI for DNA (blue). Arrows in the DAPI panel show nuclei linked by chromatin bridges. Bar 20 µm, scale is the same in all panels.
(TIF)

Table S1 GC/MS analysis of different peaks in the GC chromatogram of fraction.
(TIF)

Table S2 Comparison of mass fragmentation between the F3.1.1 peak at 68.43 min, incensole, incensole oxide and cembrene.
(TIF)
