# Supplementary figures and images for: Potent Antiproliferative Cembrenoids Accumulate in Tobacco upon Infection with Rhodococcus fascians and Trigger Unusual Microtubule Dynamics in Human Glioblastoma Cells
Source: PLoS One. 2013 Oct 22;8(10):e77529. doi: 10.1371/journal.pone.0077529 (PMC3805576; doi:10.1371/journal.pone.0077529)

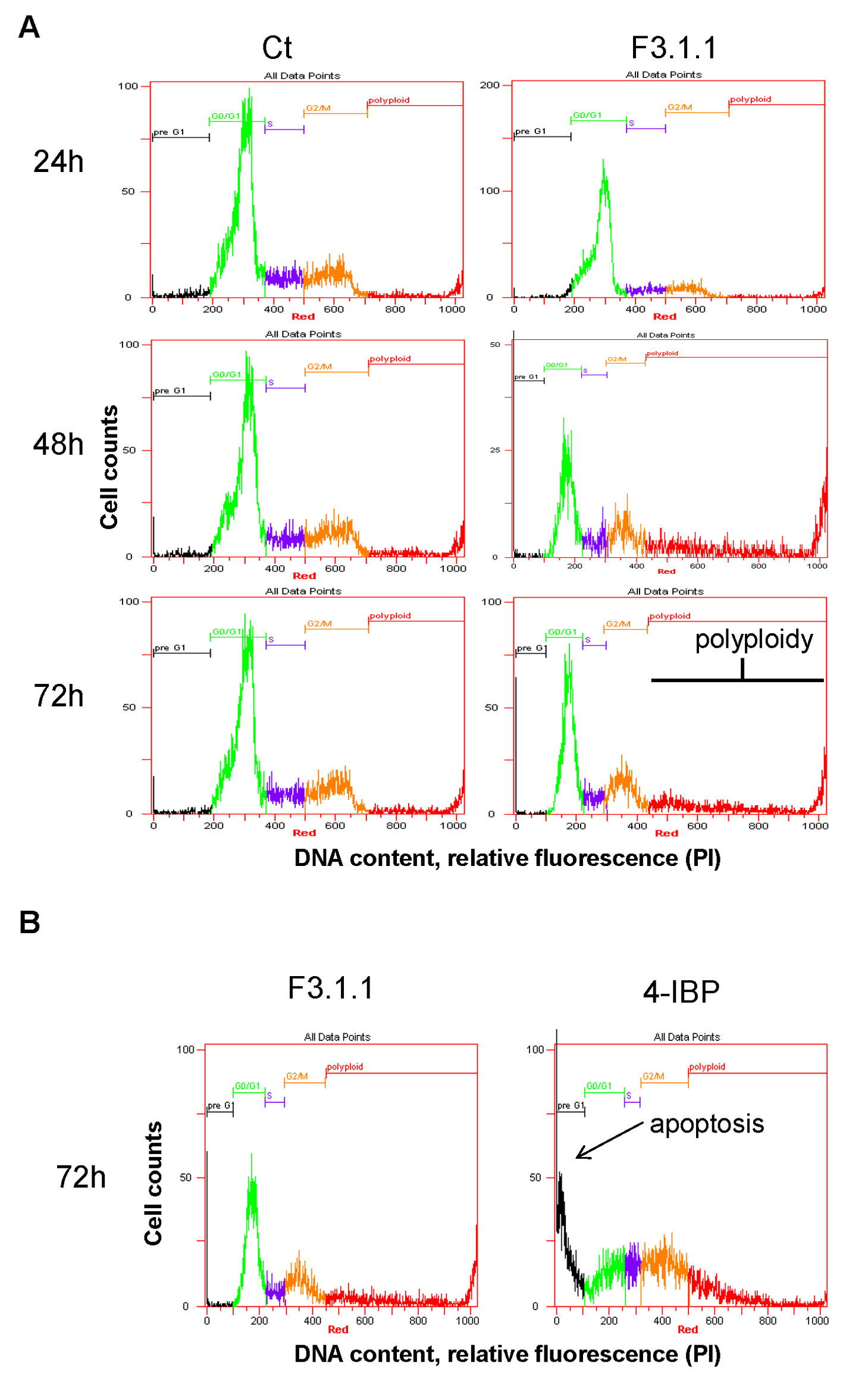

Supplement: Figure S1 — Flow cytometry analysis of the relative cell cycle phase distribution of treated and control U373 cell cultures. (A) Control (Ct) or treated cells with F3.1.1 at 4 µg/mL (n = 3); (B) Apoptosis induced in cells treated with 4-IBP at 10 µM (n = 3). (TIF) [file pone.0077529.s001.tif]

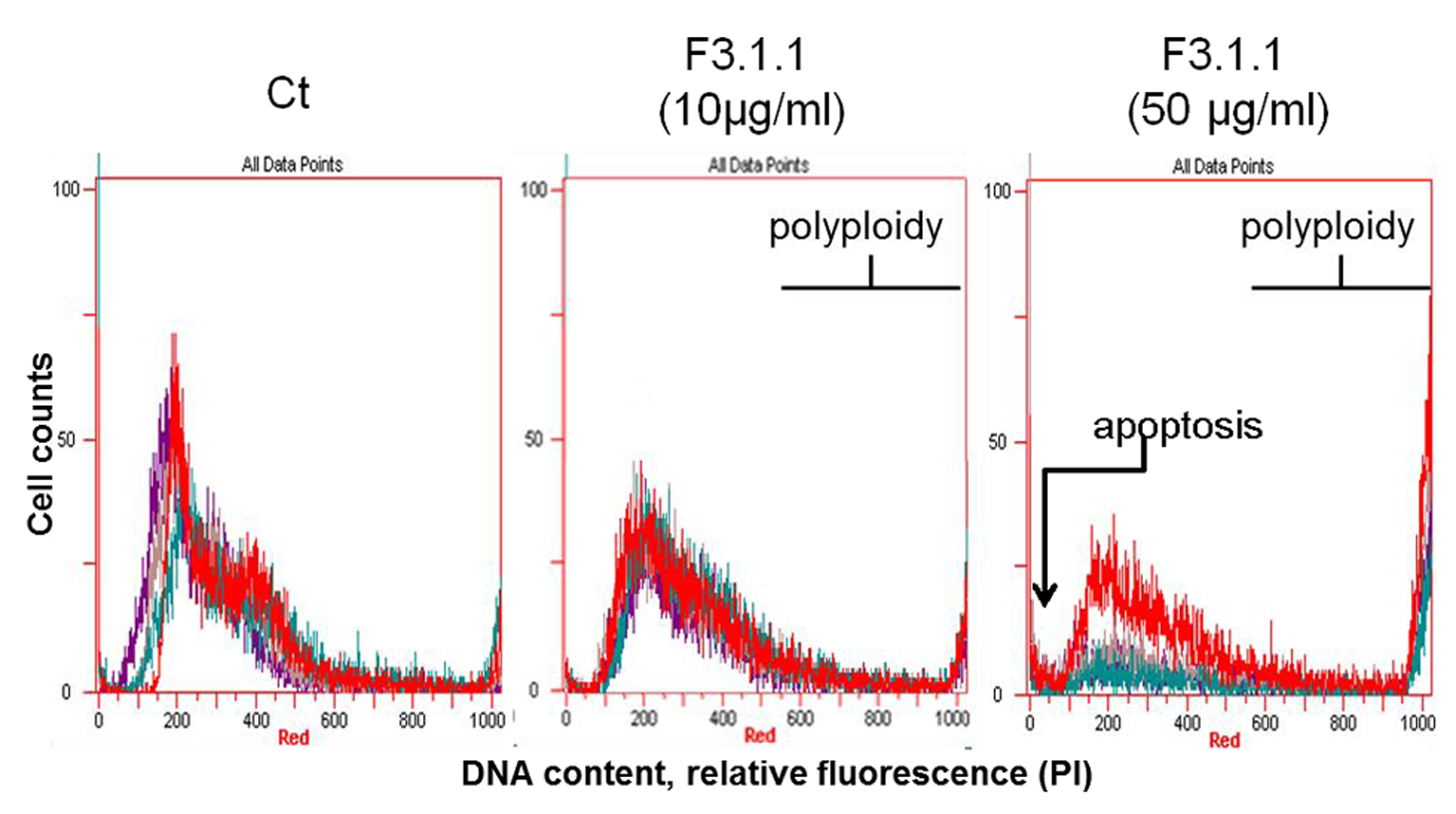

Supplement: Figure S2 — Flow cytometry analysis of U373 cells treated with higher concentrations of F3.1.1. As compared to control cells (Ct), a complete cell cycle disruption is observed with 10 µg/mL and 50 µg/mL of F3.1.1. A concomitant increase in polyploidy is observed in cells treated with 50 µg/mL of F3.1.1 (overlap of four experiments n = 4). (TIF) [file pone.0077529.s002.tif]

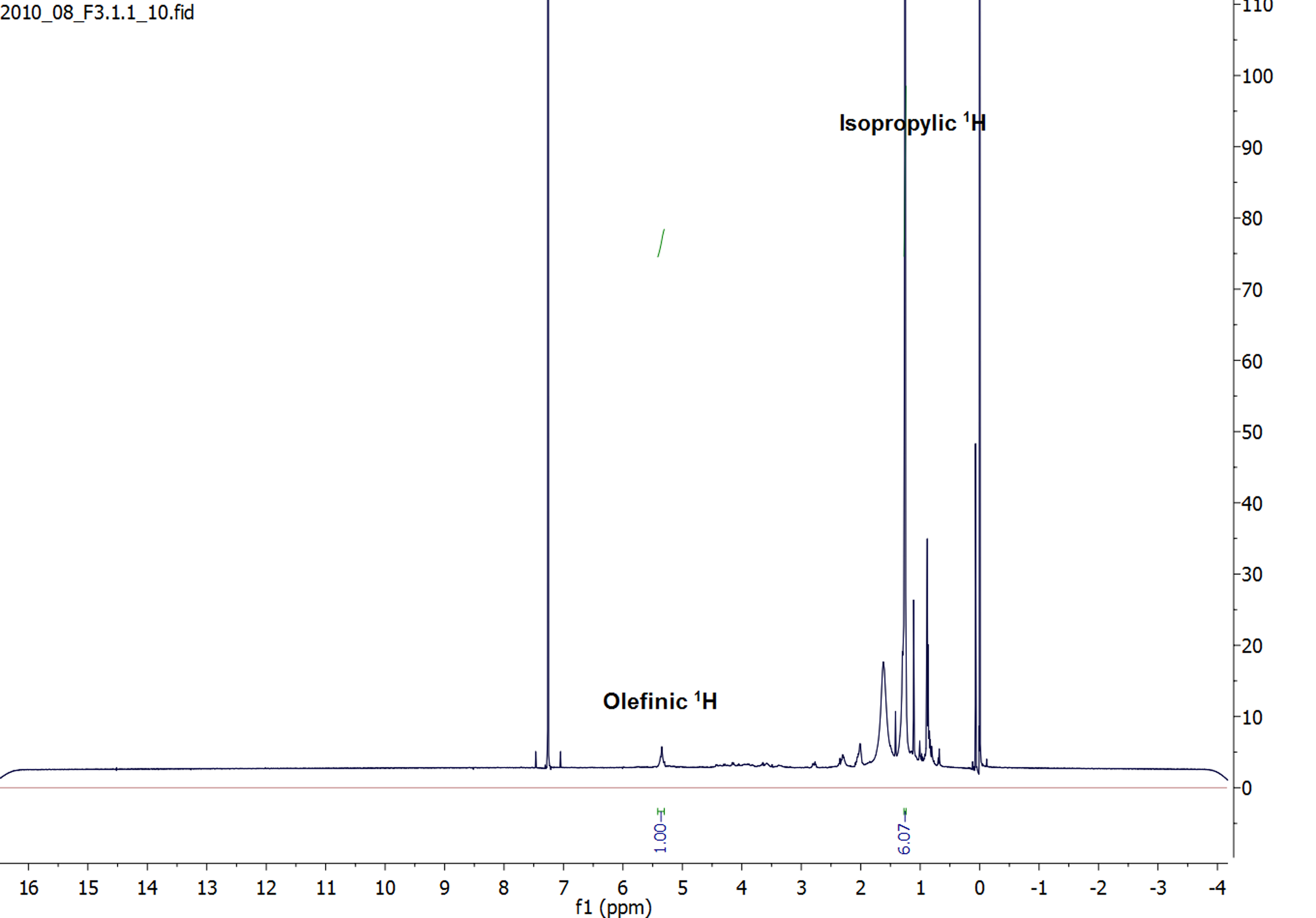

Supplement: Figure S3 — 1H NMR spectrum of the F3.1.1 mixture. Proton chemical shift at δ 5.35 ppm and δ 1.27 ppm correspond to double bonds and isopropyl groups respectively. 1H NMR (CDCl3, 500 MHz, pulse width of 5 µs (flip angle of 30°), scans number of 32, Bruker Avance II, TMS). (TIF) [file pone.0077529.s003.tif]

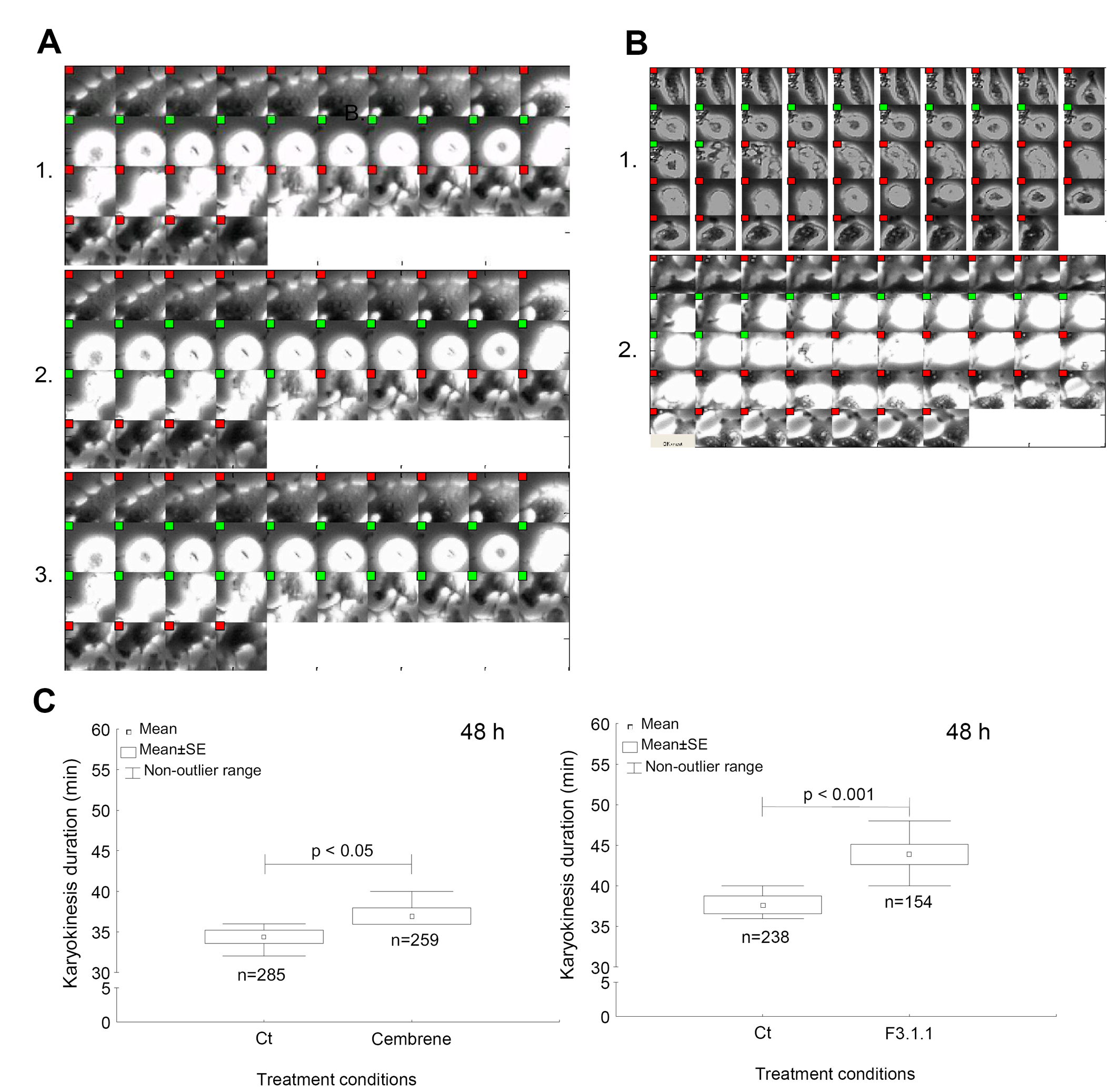

Supplement: Figure S4 — Analysis of videomicroscopic image sequences and karyokinesis duration in control and treated U373 cells. (A) Triplicate of a representative image sequence of a dividing control cell with the green squares in the thumbnails indicating that part of the sequence that identifies a particular event such as (1) karyokinesis, (2) division with well-defined daughters, and (3) cytokinesis. (B) Two image sequences illustrating karyokinesis defects in cembrenoid-treated cells with the green squares in the thumbnails indicating that part of the sequence that identifies (1) the inability to distinguish cytokinesis, and (2) the inability to distinguish two daughter cells due to asynchronous division. (C) Average cell karyokinesis duration over a time period of 48 h in control (Ct) and treated cells with 4 µg/mL cembrene or F.3.1.1. The number of cells undergoing karyokinesis is indicated (n). The statistical significance was evaluated as described in Methods. (TIF) [file pone.0077529.s004.tif]

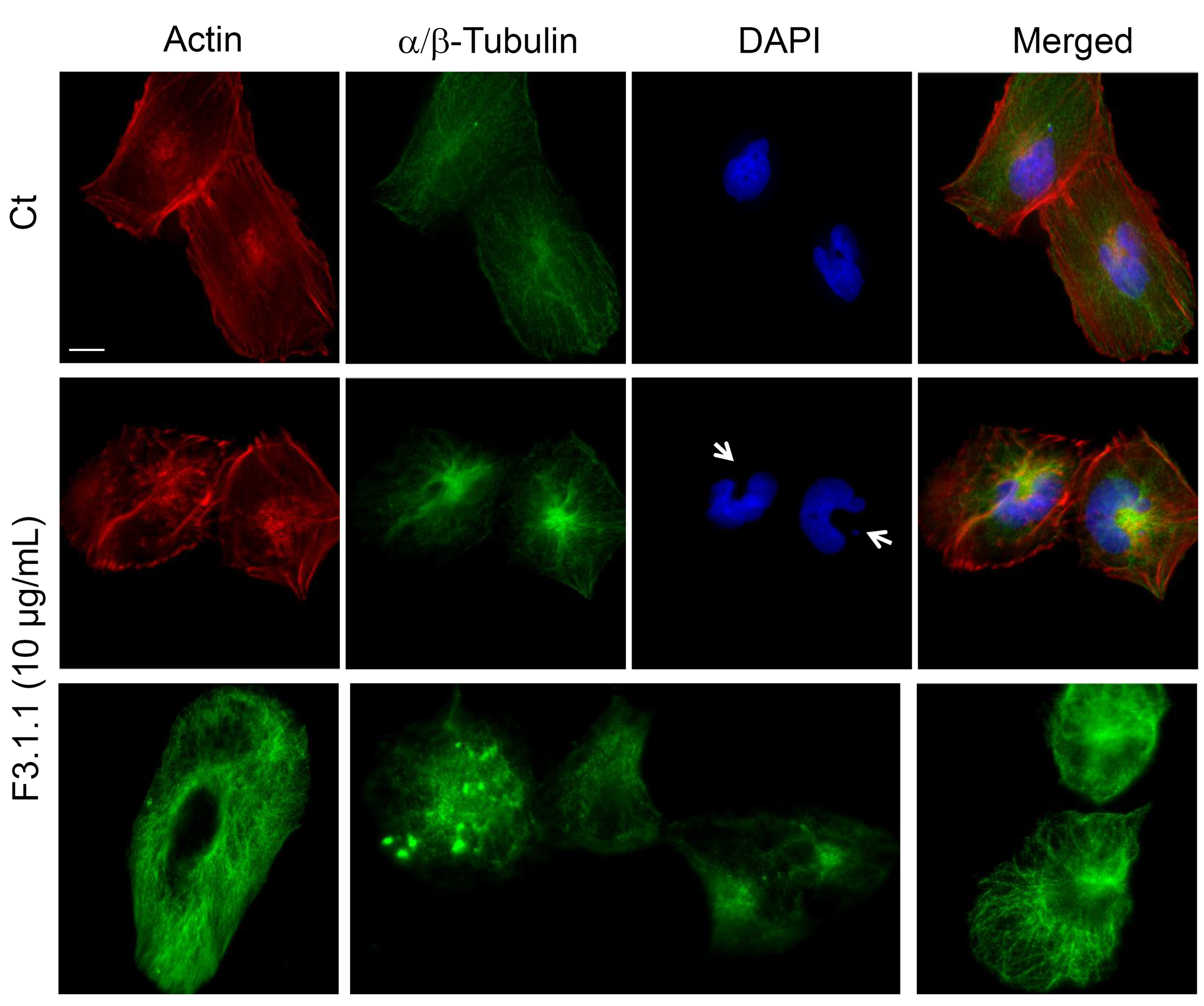

Supplement: Figure S5 — Actin, tubulin and DNA visualization in T98G cells upon treatment with 10 µg/mL F3.1.1. Immunostaining of T98G cells untreated (Ct) and treated with F3.1.1 (2×IC50) for 24 h. Cells on coverslips were fixed and stained with anti-a/b-tubulin, followed by phalloidin-conjugated to Alexa Fluor 488 and finally with DAPI. Arrows show abnormal nuclei. Bar 20 µm, scale is the same in all panels. (TIF) [file pone.0077529.s005.tif]

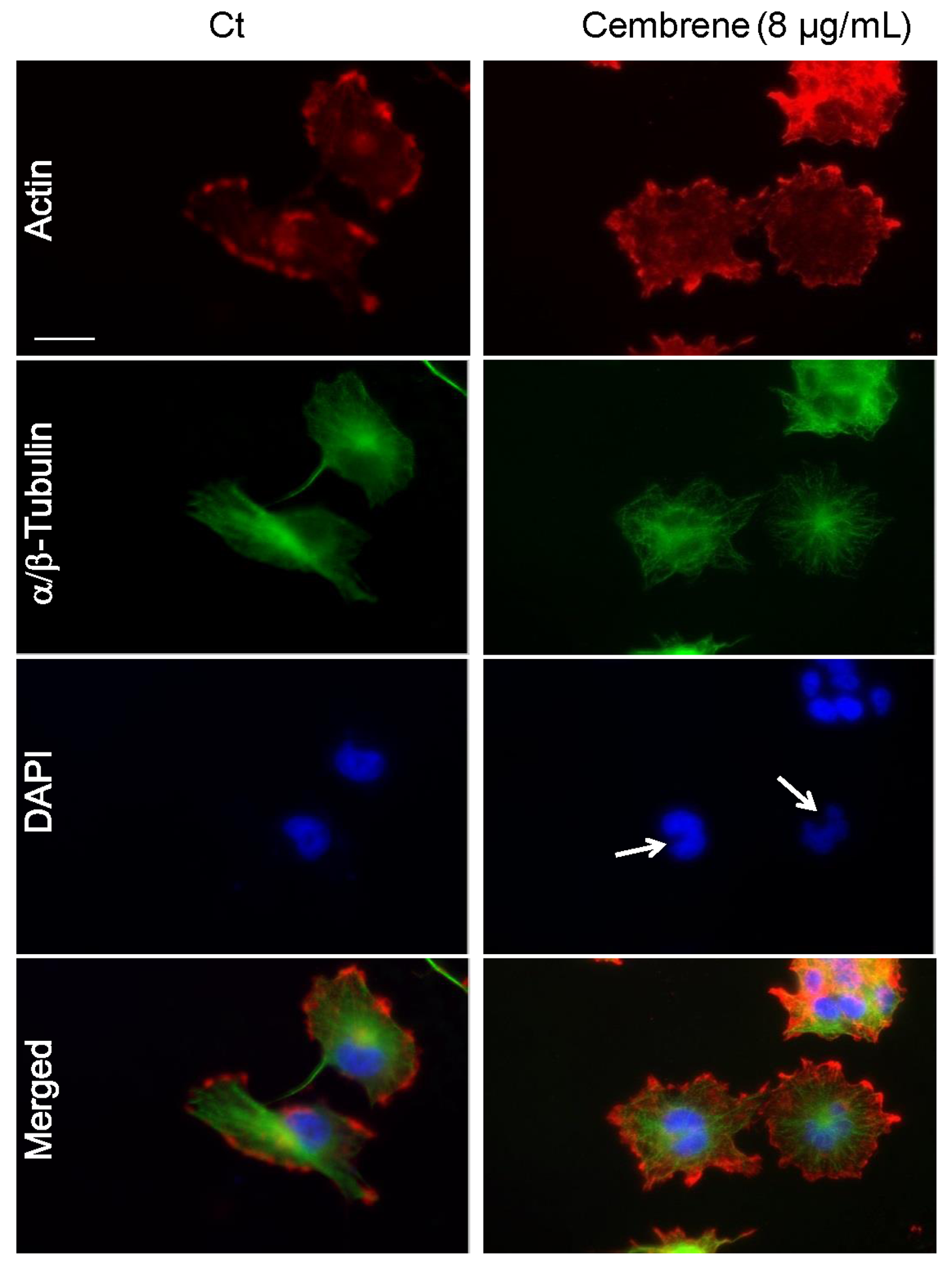

Supplement: Figure S6 — Actin, tubulin and DNA visualization in U373 cells upon treatment with 8 µg/mL cembrene. Immunostaining of untreated (Ct) and treated cells (2×IC50) after 24 h of incubation. Cells on coverslips were fixed and stained with anti-α/β-tubulin antibodies for tubulin (green), followed by phalloidin conjugated to Alexa Fluor 488 for actin (red) and/or with DAPI for DNA (blue). Arrows in the DAPI panel show nuclei linked by chromatin bridges. Bar 20 µm, scale is the same in all panels. (TIF) [file pone.0077529.s006.tif]

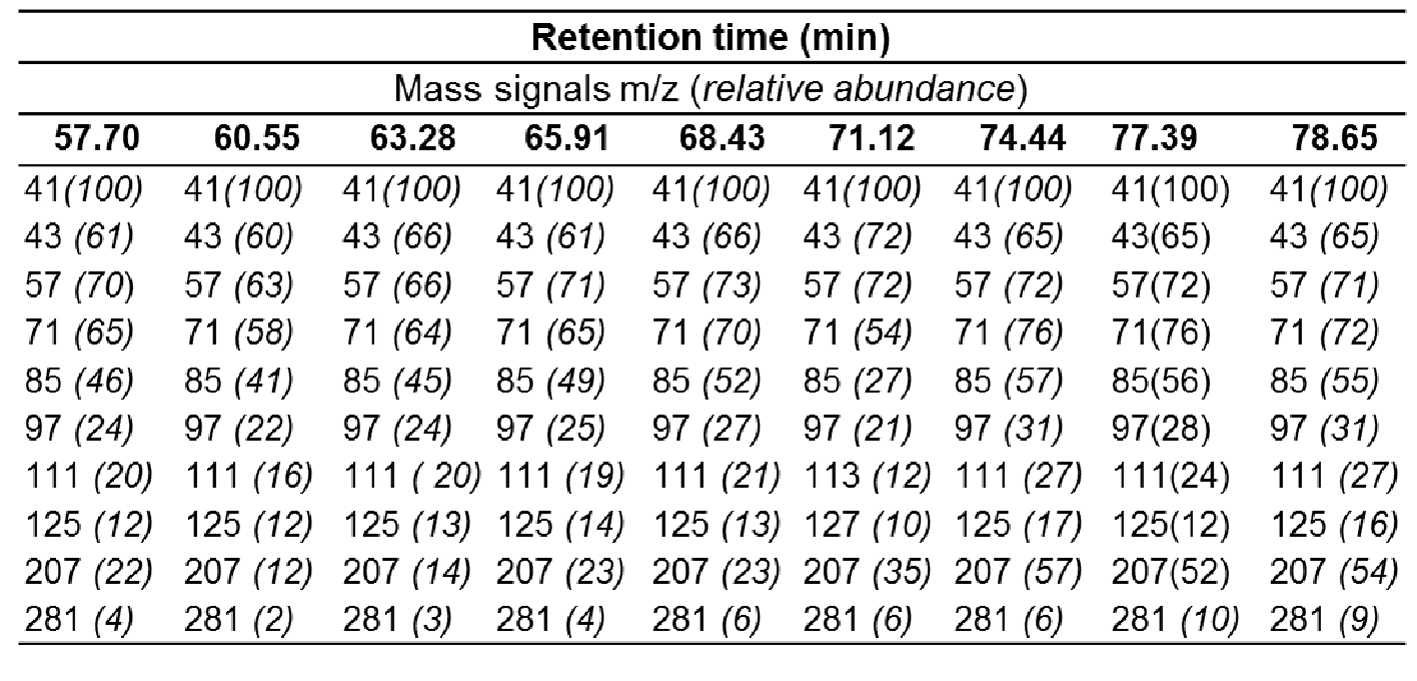

Supplement: Table S1 — GC/MS analysis of different peaks in the GC chromatogram of fraction. (TIF) [file pone.0077529.s007.tif]

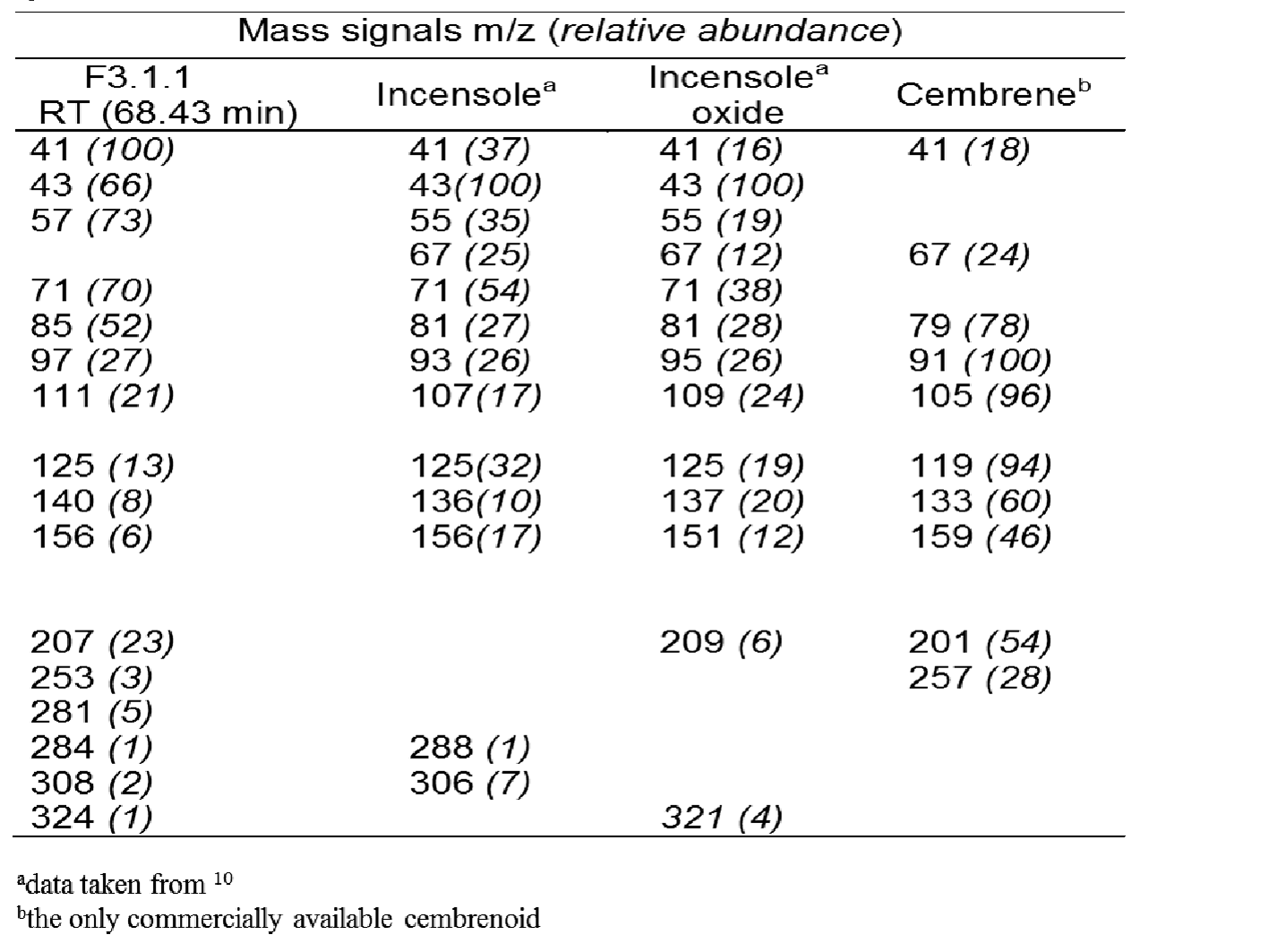

Supplement: Table S2 — Comparison of mass fragmentation between the F3.1.1 peak at 68.43 min, incensole, incensole oxide and cembrene. (TIF) [file pone.0077529.s008.tif]
